# 3D Sensors for Sewer Inspection: A Quantitative Review and Analysis

**DOI:** 10.3390/s21072553

**Published:** 2021-04-06

**Authors:** Chris H. Bahnsen, Anders S. Johansen, Mark P. Philipsen, Jesper W. Henriksen, Kamal Nasrollahi, Thomas B. Moeslund

**Affiliations:** Visual Analysis and Perception (VAP) Lab, Aalborg University, Rendsburggade 14, 9000 Aalborg, Denmark; asjo@create.aau.dk (A.S.J.); mpph@create.aau.dk (M.P.P.); jwhe@create.aau.dk (J.W.H.); kn@create.aau.dk (K.N.); tbm@create.aau.dk (T.B.M.)

**Keywords:** sewer inspection, sewer pipes, 3D vision, 3D reconstruction, computer vision, automated inspection

## Abstract

Automating inspection of critical infrastructure such as sewer systems will help utilities optimize maintenance and replacement schedules. The current inspection process consists of manual reviews of video as an operator controls a sewer inspection vehicle remotely. The process is slow, labor-intensive, and expensive and presents a huge potential for automation. With this work, we address a central component of the next generation of robotic inspection of sewers, namely the choice of 3D sensing technology. We investigate three prominent techniques for 3D vision: passive stereo, active stereo, and time-of-flight (ToF). The Realsense D435 camera is chosen as the representative of the first two techniques wheres the PMD CamBoard pico flexx represents ToF. The 3D reconstruction performance of the sensors is assessed in both a laboratory setup and in an outdoor above-ground setup. The acquired point clouds from the sensors are compared with reference 3D models using the cloud-to-mesh metric. The reconstruction performance of the sensors is tested with respect to different illuminance levels and different levels of water in the pipes. The results of the tests show that the ToF-based point cloud from the pico flexx is superior to the output of the active and passive stereo cameras.

## 1. Introduction

Sewer networks are a critical piece of infrastructure that enable the proper disposal of wastewater and rain water in modern societies. Even though the uphold of sewers is instrumental to our modern way of living, their location in the underground means that the assessment and maintenance of the sewer infrastructure is often overlooked or postponed. The ubiquitous nature of sewers is confirmed by the raw numbers; in the US, the extent of the public sewer network are more than 1.2 million km [1] and in Germany, there are more than 594,000 km of sewage and rain water pipes [2]. As the sewer pipes age, so does the likelihood of damage or leakage of the pipes. In order to prevent an untimely breakdown and replacement of a pipe, rehabilitation works may be carried out on older networks. As more than 70,000 km of the German sewage pipes were built before 1960 [2], there is a need for timely inspection of the sewer network in order to assess the condition of the pipes. However, approximately 14% of the pipes are not accessible with current inspection techniques [3] and only 13% of the responding authorities in a German survey can approve the current state of their sewer systems [3]. There is thus a need for further rehabilitation and renovation works in the sewer systems. The allocation of resources for such works is guided by inspections of the current state of the sewer systems.

Currently, sewer inspections are carried out by trained professionals who manually operate a custom-built tethered inspection vehicle that is inserted from a well into the sewer pipe [4]. The human operator controls the vehicle remotely from above ground and manually annotates the conditions of the pipe by inspecting the Closed-Circuit Television (CCTV)-footage that is streamed from the vehicle. In order to decrease the risk of the tether getting stuck in the pipe, the inspection vehicle only drives from one well to another before being extracted and inserted into the next well. Effort has been put into automating parts of the process such as the automatic locomotion of the robot [5] or the automatic annotation of the image data provided by the CCTV-footage of the robot [6].

The vision of a fully autonomous robot for sewer inspection has spurred many interesting research projects over the last 25 years which have produced several robotic prototypes for demonstration purposes such as KURT [7], MAKRO [8], KANTARO [9], ARSI [10], and SIAR [11]. For a full overview, we refer to the excellent survey by Mirats-Tur and Garthwaite [12]. The focus of the research projects listed above is mainly on the challenging task of building a robotic prototype that can navigate within the confined space of a sewer network whereas less attention has been devoted into the design and test of the sensing capabilities of said robots. There is a need, however, to study the proper selection of sensors that should guide the autonomous sewer inspection robots of the future. As evidenced by Haurum and Moeslund [6], there is increasing interest in the research community for automating the annotation process of the images extracted from the CCTV-footage of the inspection vehicles.

However, a fully autonomous, untethered robot faces a tough navigation challenge when it must traverse the entire sewer system of a neighbourhood or an entire city. This includes keeping track of the position of the robot in an Global Navigation Satellite System (GNSS)-denied environment and safely maneuvering the robot in a hostile environment. It is therefore necessary to move from two-dimensional image-based inspection towards three-dimensional (3D) depth-based inspection guided by guided by recent advances in robust, low-powered depth sensors. Although such sensors have been applied in recent projects on autonomous sewer inspection robots [10,11], their depth sensing qualities have not been thoroughly assessed.

The focus of this work is to study the characteristics of 3D sensors that can be attached on a future autonomous robotic platform for sewer inspection. In particular, we study how the choice of 3D sensors affect the performance of 3D reconstruction of the sewer pipe which is important for the condition assessment of the sewer pipe and navigation and localization capabilities of the autonomous robot. An autonomous robot should not be hindered by any tethering to the surroundings. However, untethered robots are constrained with respect to battery power and capacity. Maintaining a sufficient lit environment in order to sense the surroundings may draw a lot of power, and it might thus be interesting to study how the presence or absence of light will impact the accuracy of the 3D sensors.

In this work, we will therefore study the influence of light on the accuracy of 3D sensors within the context of sewer inspection. Furthermore, we will study how the presence of water in the sewer pipes affects the capabilities of depth sensors. Our contributions are as follows:

- We simulate the sewer environment using two different setups: a clean laboratory environment with reflective plastic pipes and an outdoor above-ground setup with four wells connected by pipes with different diameters and topology.
- We utilize the laboratory and above-ground setup to systematically test passive stereo, active stereo, and time-of-flight 3D sensing technologies under a range of different illuminance levels.
- The laboratory setup is utilized for assessing how active stereo and time-of-flight sensors are affected by the presence of various levels of water in the sewer pipe.
- We systematically evaluate the reconstruction performance of the 3D sensors from the experiments and compare it with reference models of the sewer pipes.

The remainder of the paper is organised as follows: Section 2 presents an overview of related work within 3D sensing of sewers and pipe-like environments. Section 3 gives an overview of suitable depth sensing technologies for inspection of sewers. Section 4 describes the experimental setup and methods used for the illumination tests and water level tests. Section 5 analyses the experimental results, and Section 6 concludes on the results and gives directions for further investigation.

## 2. Related Work

The need to autonomously navigate, map and inspect hazardous environments has in recent years led to increasing interest for implementing 3D vision techniques to create a dense representation of the surroundings. On the MAKRO robot prototype in the late 90s, a laser projector is combined with a rotating mirror to map the distances to the upper sections of the sewer pipe [13]. A similar method is used on the KANTARO robot where the infrared range scanner is located on a protruding stick at the back of the robot [9].

In order to map the scope of 3D vision techniques for compact environments, a systematic literature study has been conducted. Seven scholarly databases have been searched for related works in the field of sewer and pipe inspection. The search was constrained to articles in which title, abstract, or keywords contained one entry from each of the terms in the list below:

- Sewer OR pipe;
- 3D;
- Inspection OR reconstruction OR assessment.

**Figure 1:**
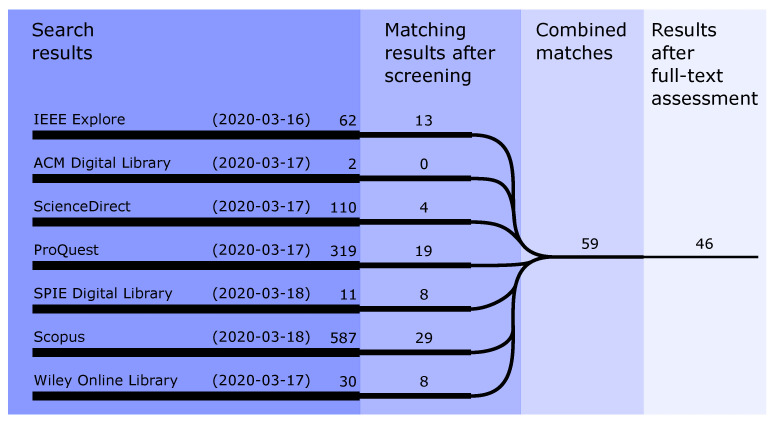
Flow of the literature search. The records of the seven scholarly databases are manually screened based on the title, abstract, and keywords. In a subsequent stage, the records are combined and checked for duplicates. In the final process (light blue), the full-text of every record is manually assessed.

The number of articles from the search of the scholarly databases is visible from the first column of Figure 1. The databases of Google Scholar and ASCE Library are excluded based on the inability to constrain the search to title, abstract, and keywords. Every record from the databases is manually screened based on the title and abstract of the record. From this stage, records that do not consider in-pipe analysis are filtered out alongside records that do not contain any sensing component. After the screening stage, the records from all the databases are combined and duplicates are removed. The full-text content of the remaining 59 articles is assessed of which 13 articles are removed based on the above criteria.

The remaining 46 articles have been assessed and categorized based on the sensors that they use to perceive the environment. The findings are summarized in Table 1 where they are located amongst other references found outside the systematic literature study. The related works are categorized into five groups in Table 1 based on the utilized depth sensing hardware.

### 2.1. Laser-Based Sensing

One of the most commonly used techniques for sensing the geometry of the pipe is by the use of laser profilometry. A rotating laser beam projects a ring inside the pipe and a camera is positioned such that it captures the full extent of the ring. By extracting the contour of the laser ring from the image, the cross section of the pipe can be measured, and any imperfections from the elliptical ring may be classified as defects or corrosion. A variant of this setup is the use of multiple fixed, non-rotating laser diodes such as in the work of Hansen et al. [43]. The positions of the laser diodes are tracked through consecutive frames, and the tracking enables estimation of the metric radius of the pipe. In [19], a crosshair laser beam is projected ahead of the inspection vehicle in order to guide the navigation and motion planning.

The conceptual simplicity of the laser-based methods is one of its virtues as a tool for an autonomous inspection robot. The cross section of the pipe is easy to interpret, and the detection of structural defects is straightforward. On the other hand, laser profilometry only scans the surface in one plane that moves along the pipe as the robot traverses. This implies that a full 3D reconstruction of the pipe is available only after the robot has moved through the entire pipe. However, an online 2D view of the pipe is not usable in order to navigate a fully autonomous inspection robot in a possibly unknown environment.

### 2.2. Omnidirectional Vision

Omnidirectional vision sensors allow for a full view of the internal surface of the pipe. The full view is either produced by a catadioptric camera system [23,44,50,55,58] or by the use of fish-eye lenses [9,25,28,29,32,53]. Omnidirectional vision sensors provide a view of the entirety of the pipe with just a single sensor and are used in combination with laser profilometry [9,23,44,50,55] or stereo matching [25,29,32,53,58] approaches.

Compared to traditional cameras, the full view comes at the cost of either complicated hardware in the case of catadioptric cameras and additional post-processing of the acquired imagery for both solutions. With careful calibration of the camera systems, however, a very detailed, wide-angle view of the pipe may be acquired.

### 2.3. Stereo Matching

A dense 3D reconstruction of the pipe may be provided by the use of stereo matching techniques with either monocular or stereo cameras. With monocular cameras, cylindrical or elliptical [25] constraints are often imposed in order to guide the reconstruction. The addition of an additional camera enables the computation of structure independent of the motion of the robot and allows for the relaxation of the cylindrical constraints. Two-view stereo matching is applied in a sewer context by Huynh et al. [45,46] to detect cracks and defects in sewers. While their experiments show that it is a viable strategy, they also note that their method has issues detecting small defects or thin cracks that are hard to locate in both camera views. A common problem with traditional passive stereo, they had problems with matching features between the two views in case of very uniform environments. Furthermore, stereo matching is a computationally heavy operation that imposes high processing requirements in order to retrieve a high-quality point cloud.

An approach to combating the feature matching problem is the use of active stereo techniques. Active stereo techniques project a set of distinct features onto the surface. The active stereo camera is combined with an Inertial Measurement Unit (IMU) for effective localization in the context of drone-based 3D cave mapping [63]. A similar approach is found in [51] for mapping of underground tunnels. The authors have replaced the active stereo depth sensor with a time-of-flight (ToF) sensor while also using monochrome Charged Coupled Device (CCD) cameras in a stereo setup. Utilizing CCD instead of Complementary metal–oxide–semiconductor (CMOS) based cameras allows them to capture images with a global shutter so light only has to be emitted from the robot the moment it takes the picture, reducing potential interference the reflected light could have on the ToF sensor.

### 2.4. Other Sensing Approaches

The recent work of Kolvenbach et al. [61] within inspection of sewers utilizes active stereo for a dense 3D map of the local environment while relying on a Velodyne VLP16 LIDAR to do a long range, rough mapping of the environment for localization and navigation. The Kinect v1 and Kinect v2 cameras are used in [57] to estimate the radius of pipes. The high-end FARO LS 840 HE laser scanner is used to build a reference model with which the much more affordable Kinect devices are compared.

## 3. Depth Sensors

Depth sensing technology is improving at a rapid pace. The number of sensors that are available has exploded while prices have reached a customer friendly level. Overall, depth sensing technologies fall into three categories: stereo vision, ToF, and depth from monocular cues.

**Figure 2:**
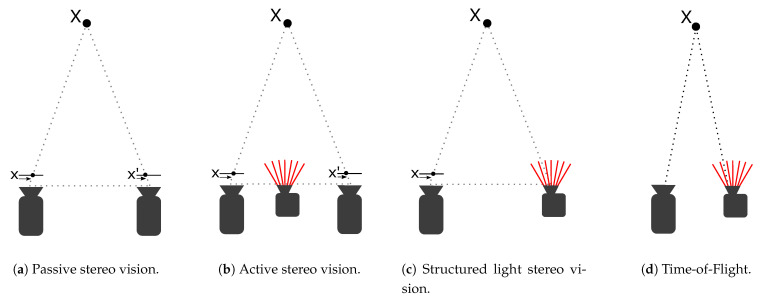
Different variations of depth sensing techniques.

Stereo-based methods rely on the parallax or displacement that occurs when an object X is viewed from different points of view. This is illustrated by pixel x and x’ in the image planes shown in Figure 2a,b, respectively. Objects close to the cameras have a greater disparity in position between the different views than objects that are further away. The depth of an object seen from both views may be computed from the disparity by the use of similar triangles and the prior knowledge of the baseline and focal length of the stereo setup. The hard problem is identifying the same locations in both views in order to calculate the disparity. Passive stereo cameras consist of two cameras that monitor the same scene from different but well known points of view. For matching algorithms to successfully identify features and calculate disparities, the scene must contain distinct features. Active stereo, as illustrated in Figure 2b, adds a projected light pattern to the scene in order to perform better in low texture environments. Otherwise, the method works in the same way as a passive stereo camera. Depth from structured light, as illustrated in Figure 2c, also projects light patterns into the scene. But instead of using two cameras, knowledge of the transmitted pattern is used to perform triangulation using a single camera and the projector. Because this method relies on recognizing a well known projected pattern, it is sensitive to external interference. All are reliant on the known relationship between the two viewpoints, making the structural integrity of the setup crucial. The three variations of stereo vision are illustrated in Figure 2.

ToF sensors measure distance based on the time it takes emitted light to be reflected by an object in the scene and return to an image sensor. This is illustrated in Figure 2d. Achieving a usable depth resolution with this method requires very high clock frequencies from the hardware. Because depth measurement does not rely on triangulation, however, ToF cameras can be made as small as the sizes of the laser and sensor allow.

Recently, advances within machine learning algorithms have shown impressive results for estimating depth from monocular vision. This is possible because visual cues such as focus, sizes, shadows, perspective, motion, etc. are correlated with depth. Since ground-truth depth data that can be used to train such algorithms is prohibitively difficult to acquire, self-supervised approaches to learning depth estimation are very enticing. Clément et al. [64] make use of the known relationship between stereo image pairs when training a CNN to estimate disparity. The right image of a stereo pair can be approximated from the left image of the same stereo pair based on the calculated disparity. Thus, a loss function can be designed to minimize the differences between the image pair given the estimated disparity by a CNN.

Monocular depth estimation can be learned using stereo pairs as described above or from monocular video. Using monocular video requires ego-motion estimates between temporal image pairs. Clément et al. [65] propose a number of tricks that improve monocular depth estimation and show how self-supervision from both video and stereo pairs can be combined. These methods may fail to correctly estimate depth for images that are distorted, contain reflections or are highly detailed.

We select the sensors for comparison in this work based on their popularity and representability of the underlying technologies. Due to the low popularity of structured light and the poor performance that is expected for this technology under less controlled conditions, structured light is eliminated up-front. It is safe to say that the Microsoft’s Kinect depth sensors have been the most widely used in 3D vision research. For this reason, Azure Kinect (Microsoft Corporation, Redmond, WA, United States), the successor to the popular Kinect 2, is an obvious candidate for our case. It is capable of acquiring accurate high resolution point clouds using ToF technology and can be optimized for a wide or narrow field of view depending on the application. The Kinect family has already been thoroughly covered by Tölgyessy et al. [66] where performance is measured across different surface types. Their research shows that surfaces at the extremes of the reflective—absorbent spectrum are most challenging. Out of the box, Azure Kinect is too fragile with an operational temperature range of 10–25 °C. Mitigating this problem will add significantly to the complexity and size of the payload. Considering the limited payload of a sewer inspection robot, PMD CamBoard pico flexx (PMD Technologies AG, Siegen, Germany), or pico flexx for short, with its small size and low weight of 8 g, is a good choice as representative of ToF sensors. It has previously been used for indoor localization and mapping in both light and dark environments [67] and for exploration and mapping of tunnels [51]. The selection of stereo cameras is large, but when limited to smaller cameras, the RealSense D400 family of stereo cameras is probably the most popular. With a short to medium size baseline and a global shutter, the RealSense D435 (Intel Corporation, Santa Clara, CA, United States)is the best choice for sewer inspection. It is used in the ARSI autonomous micro air vehicle for inspection of sewers [10] and for aerial robotic navigation in low–light GNSS–denied conditions [68]. Because it contains a NIR laser projector it can function as either a passive or active stereo camera and lets us cover both passive and active stereo vision using a single camera. Table 2 lists some of the central specifications of the two cameras.

## 4. Materials and Methods

The Realsense and pico flexx depth sensors that have been selected as representatives of the different relevant depth sensing technologies and as potential sensors for a sewer inspection robot platform are tested in order to assess and compare their suitability for inspection of sewer pipes.

The tests serve two purposes, namely, to investigate the sensitivity of the sensors with respect to

- Different illuminance levels in the pipe;
- Different water levels in the pipe.

The illumination tests are conducted both in a laboratory and in an above-ground outdoor test setup whereas the water level test is conducted solely in the laboratory.

### 4.1. Robotic Setup

The depth sensors are integrated on a purpose-built LEGO platform and connected to a Raspberry Pi 4 with 4 GB of RAM (Raspberry Pi Foundation, Cambridge, United Kingdom). Sensor data are recorded directly on the Raspberry Pi and subsequently transferred to a PC for analysis.

**Figure 3:**
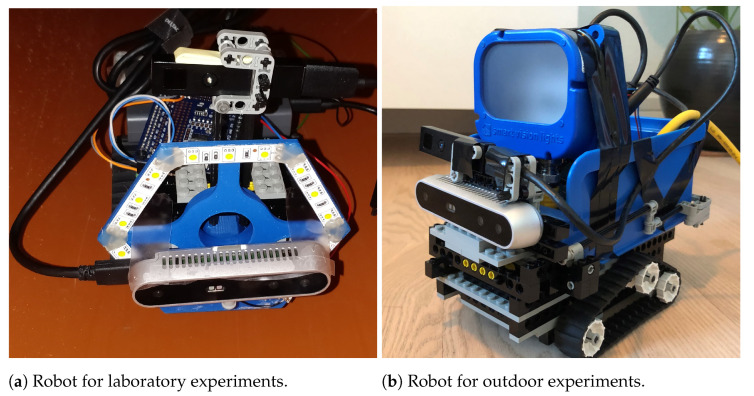
Robotic setup for the laboratory and outdoor experiments. The setup is upgraded for the outdoor experiments with a new light source and a blue box that protects the Raspberry Pi from dirt.

The illumination of the sewer pipe is provided by two different light sources; a custom-built ring light for the laboratory experiments and an industrial-grade brick light [71] for the outdoor experiments. The robotic setups for both cases are shown in Figure 3.

As noted in Section 3, the Realsense camera contains an infrared emitter that can be turned on and off. When turned off, the Realsense works as a traditional passive depth sensor, relying only on the ambient light for estimating the depth of objects. When the infrared emitter is turned on, the camera may operate without any external light source. These two modes of operation are tested for the illumination tests whereas only the default mode with the emitter turned on is tested for the water level experiments. Any other setting on the Realsense camera is set as default. The pico flexx camera comes with a variety of work modes. For these experiments, the camera is configured with “Mode 9” that enables a frame rate of 15 frames/second, which results in a reported depth range of 0.5–1.5 m [72]. The configuration is a trade off between range, accuracy, and frame rate. It is assumed that the sewer robot will move slowly in the sewer pipes, which means that a frame rate of 15 frames/second is sufficient. The chosen configuration is the one that matches an acceptable accuracy with a minimum depth range that allows the robot to see objects within a reasonable driving range.

### 4.2. Assessing the Point Clouds

**Figure 4:**
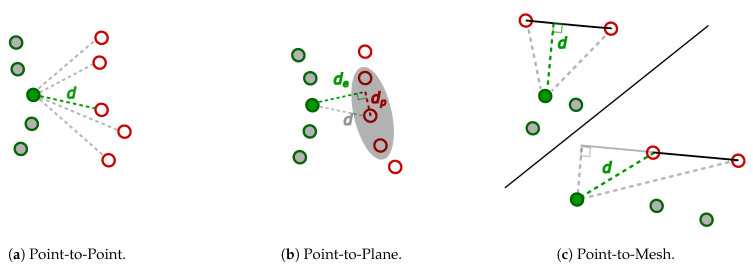
Three different types of point cloud comparison. Red markings signify the reference, while green and gray signify the measured object.

The raw output of the illumination tests and water level tests is a sequence of point clouds from each sensor. Methods for assessing the quality of the point clouds may be grouped into two broad categories: quantitative analysis by comparing the point clouds with a reference model and human perception for judging the visual traits of the point clouds. The following provides an overview of techniques for performing quantitative analysis of point clouds:

**Point-to-Point**  The distance to the nearest neighbor is found by measuring distances between a given point and every point in a reference point cloud. This is illustrated in Figure 4a where the closest point in the reference point cloud is found, and distance *d* corresponds to the matching error for the point pair marked with green.
**Point-to-Plane**  The method estimates the surface of the reference point cloud. The surface of a point in the reference cloud is computed using the neighborhood points around the matching point in the reference cloud. In Figure 4b, the neighborhood is illustrated by the gray oval, and the estimated surface is represented by the dotted red line. The vector between the point-to-point match connected by *d* is projected onto the surface of the reference cloud. Together, the point-to-point vector *d* and the vector $d_{p}$ projected onto the reference surface are used to compute an error vector $d_{e}$ that is normal to the estimated surface in the reference cloud [73].
**Point-to-Mesh**  Distances between a point cloud and a reference mesh are defined as either the orthogonal distance from a point in the measured cloud to the triangular plane in the reference mesh or as the distance is to the nearest edge, in case the orthogonal projection of the point falls outside the triangle [74]. The two possibilities are illustrated in Figure 4c.

Alternatively, human perception can be used to compare and judge point clouds [75]. This requires time and calibration and is likely to produce highly subjective results. The approach is well suited for cases where the point cloud is intended for human consumption or when an algorithmic approach is not feasible. Besides its uses in evaluation of data acquisition systems, comparison and quality assessment of point clouds is used in the evaluation of denoising [73] and compression [76] methods and used in methods for measuring of terrestrial surface changes due to erosion, sedimentation, growth, and construction [77].

**Figure 5:**
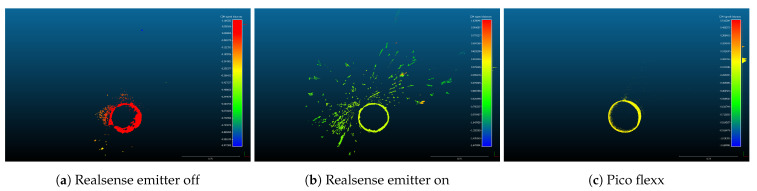
Cloud-to-mesh distances from the depth sensor to the 3D model (not shown). Screenshots from CloudCompare [78]. Please note that the color scale vary between the plots.

As stated in the Introduction, the point clouds generated from the depth sensors should assist an autonomous inspection robot in reconstructing the sewer pipe in order to detect defects, map the topology of the pipe, and determine drivable sections of the pipe. In order to complete these tasks, it is important that the acquired point cloud be an accurate representation of the real pipe. As the experiments are formed in mostly flush pipes, it is a fair approximation to model the pipe as a geometric cylinder with a diameter that resembles the interior diameter of the pipe. The geometric cylinder as a reference model also provides a mesh that can be used for computing distances from the cylindrical surface to the point cloud. This enables the use of the point-to-mesh method mentioned above. The comparison of the acquired point cloud to the reference mesh of a cylinder is performed as follows:

1. A point cloud is constructed from each depth image by back-projecting the depth points by the use of the intrinsic parameters from each camera.
2. A 3D model of the sewer pipe is loaded into the CloudCompare software toolbox [78]. The 3D model is made such that it matches the interior diameter of the sewer pipe.
3. The point cloud of each sensor is loaded into CloudCompare and cropped if necessary.
4. The loaded point cloud from the sensors are likely displaced and rotated with respect to the 3D model which means that they cannot be directly compared. There are two methods for aligning the point cloud to the 3D model:
  (a) Directly measuring the rotation and displacement of the depth sensor with respect to the pipe.
  (b) Estimating the transformation by proxy methods that works directly on the point cloud.
5. For these experiments, method (b) is chosen as it was not possible to directly measure the position of the sensor with the required accuracy. A RANSAC-based method [79] available as a plug-in to CloudCompare is used to estimate cylinders from the point cloud of each sensor. If the reconstructed point cloud is noisy, the RANSAC estimation might produce several cylinders. The cylinder that resembles the physical pipe is selected manually. The affine transformation that relates the cylinder to the origin of the coordinate frame is obtained.
6. The inverse of the transformation obtained from step 5 is used to align the point cloud to the 3D model, assuming that the 3D model is centered at (0,0,0), that the circular cross-section of the cylindrical model is parallel to the XY-plane, and that the height of the cylinder is expanding along the *z*-axis.
7. The distance between the point cloud from the sensor and the 3D model is computed by using the “Cloud to mesh distance” tool from CloudCompare. The distance is stored as a scalar field in a new point cloud. Sample distance measurements from the Realsense and pico flexx depth sensors are shown in Figure 5.

### 4.3. Illumination Tests

**Figure 6:**
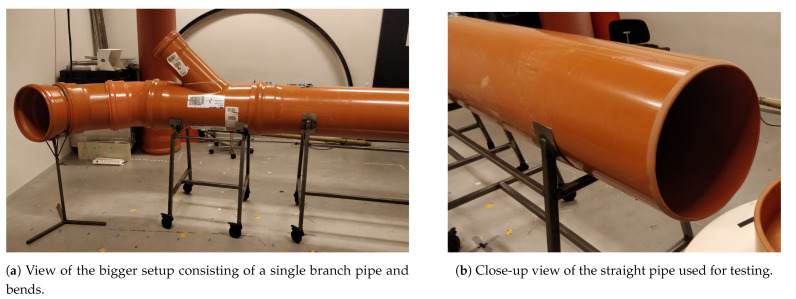
Laboratory experimental setup.

**Figure 7:**
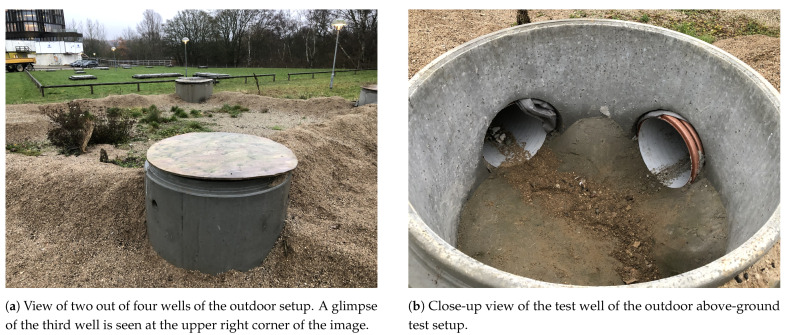
Outdoor above-ground experimental setup. The four wells are laid out in a square configuration with 5m between each corner.

The sensors are tested in two test setups under a range of illumination levels. The setups are constructed as follows:

1. A university laboratory containing no windows in order to emulate the complete darkness of certain sections of a sewer pipe. A standard Ø400 mm PVC pipe with a length of 2 m is located on top of a metal frame that enables easy access for scientific experimentation. The setup is shown in Figure 6.
2. An above-ground outdoor test setup consisting of four wells connected by Ø200 mm and Ø400 mm PVC pipes. Each well may be covered by a wooden top to prevent direct sunlight from entering the pipe. The pipes are laid out in different configurations to simulate straight and curved pipes, branch pipes, and transitions between pipes of different materials and diameters. The pipes are covered with sand to block to block sunlight from entering the pipes and to enable rapid manipulation of the configuration of the pipes. The above-ground setup is pictured in Figure 7.

The depth sensors are tested through a wide variety of illumination levels enabled by adjusting the input voltage of the light sources. The intensity of the illumination is measured with a light meter [80] that is positioned with a distance of 40 cm from the light source. The laboratory experiments consist of 23 illuminance levels ranging from 0.02 lx to 294 lx whereas outdoor experiments consist of 9 illuminance levels ranging from 1.93 lx to 619 lx. The finer granularity of the laboratory experiments enable the study of the importance of illumination to the depth sensors in finer detail while the higher illumination level from the outdoor experiments will reveal if the sensors benefit from even more light than present in the laboratory experiments. The details of the experimental setups are found in Table 3.

### 4.4. Water Level Tests

**Figure 8:**
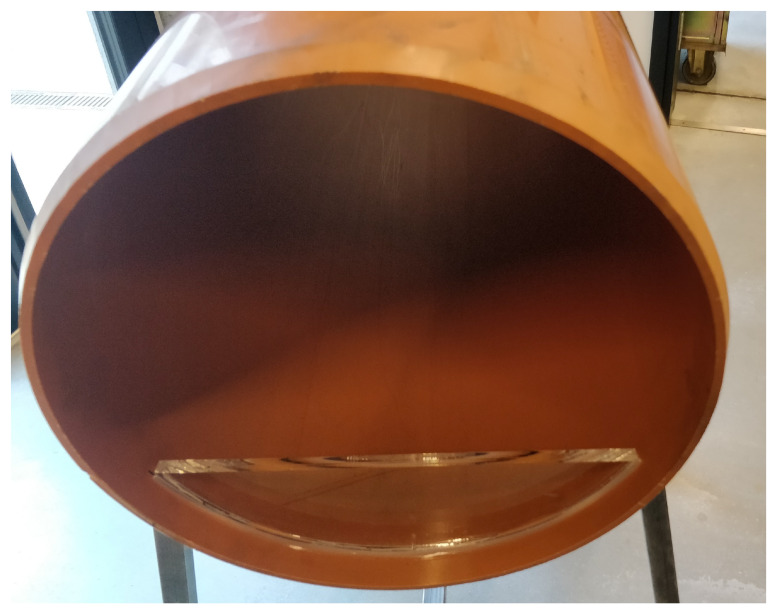
Water level setup. A piece of acrylic glass is inserted at both ends of the pipe in order to accommodate water.

**Figure 9:**
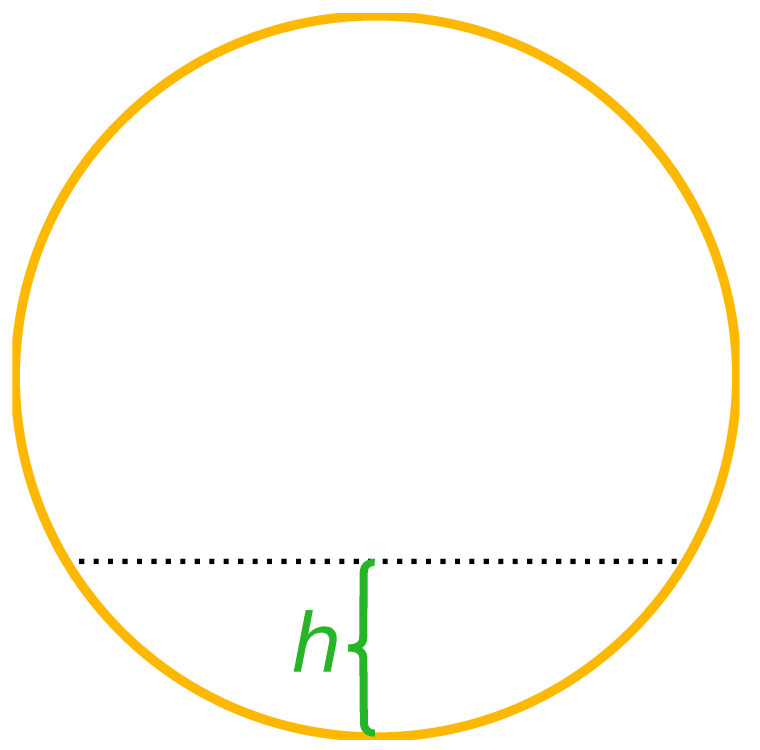
Cross-sectional view of the sewer pipe. The water level is measured by *h* which is the line orthogonal to the water line and the bottom of the pipe.

The laboratory setup is reconfigured to allow for water to accumulate in the pipe. A piece of acrylic glass is inserted at both ends of the sewer pipe and fastened with glue as seen from Figure 8. The acrylic glass allows for water levels in the pipe between 0 and 80 mm. The water level is measured as the distance between the surface of the water and the bottom of the pipe, illustrated in Figure 9. For the experiments, the water level is varied in steps of 20 mm from 0 to 80 mm. In order to emulate the dirty water flowing through the real sewer pipes, we pollute clean tap water from the laboratory by adding sand, milk, and ground coffee. The small amount of sand models sediments in the pipes and the addition of milk and coffee renders the water opaque and cloudy. Details of the pollutants are found in Table 4. The reference 3D model described in Section 4.2 is modified to account for the different water levels of the experiments. For each water level experiment, a plane is injected into the 3D model that resembles the specific water line of the experiment. This construction entails that a point cloud from a sensor is not penalized if the point cloud contains points lying on the water line or contains points lying on the pipe floor below the water line.

## 5. Experimental Results

The results of the experimental tests of the illumination levels and water levels are described in the following.

### 5.1. Illumination Tests

**Figure 10:**
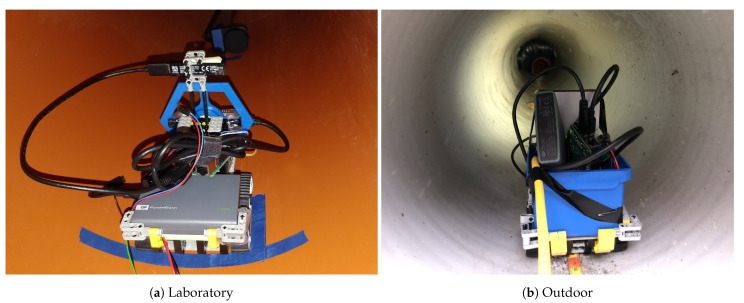
View of the LEGO-based robot located in the laboratory (**a**) and outdoor (**b**) pipes. The lux meter is visible in the top of image (**a**). The lux meter was not present during the recording of sensor data.

The illumination tests are conducted by placing the LEGO-based robot inside the pipe such that the sensors and light source are placed at least 0.2 m inside the pipe. The placement of the robot inside the laboratory and outdoor pipes is seen in Figure 10. The input voltage to the light source is regulated in steps that adhere to the specifications from Table 3. After each adjustment of the input voltage, external light is blocked from the scene. In the laboratory setting, this is achieved by turning off the lights in the windowless room and in the outdoor setting, wooden covers are placed on top of the wells. The depth sensors are hereafter initialized and instructed to record footage of the scene for 5 to 10 s. The sensor recordings are post-processed offline after the protocol described in Section 4.2. In step 3 of the post-processing, the point clouds are cropped such that −2 m≤x≤2 m, −2 m≤y≤2 m, and 0 m≤z≤2.5 m. The *x* and *y* range is deliberately chosen to be very permissive such that a robot would be able to drive in very large pipes without any assumptions. The z range is chosen due to the maximum extent of the investigated pipes.

**Figure 11:**
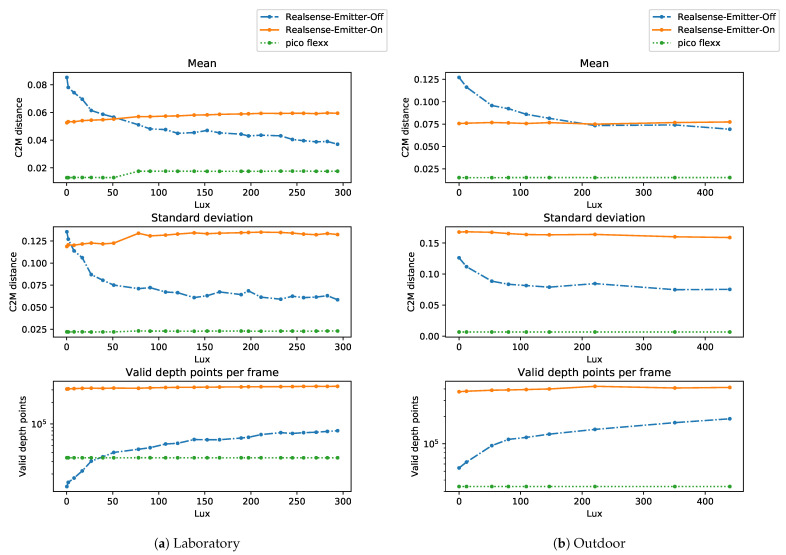
Mean (**top**), standard deviation (**middle**) of the reconstruction error, and (**bottom**), average number of valid depth points from each depth image of the camera. C2M distance: cloud to mesh distance.

The aggregated results of the overall reconstruction performance with respect to the amount of light available in the pipes are shown in Figure 11. From the first glance at the results from Figure 11, the pico flexx camera seems superior to both Realsense configurations in terms of both mean average error and standard deviation. The performance of the pico flexx is, as expected, not affected by the absence or presence of visual light in the pipes.

This is a different story for the Realsense cameras. In the following, we will use the following short names for the Realsense: Realsense-Off for the Realsense camera with the infrared emitter turned off and Realsense-On for the Realsense camera with the infrared emitter turned on. Under low-light conditions, the Realsense-On camera is the better Realsense camera. Surprisingly, however, is the observation that the reconstruction performance of the Realsense-On camera does not improve with the addition of more light in the scene. On the contrary, the mean reconstruction error seems to slightly increase with the addition of more light. This suggests that the Realsense-On camera almost entirely relies on the reflections from the infrared dot projector.

On the other hand, the Realsense-Off camera benefits immensely from the presence of infrared and visual light. This is expected, as the camera in this configuration is a passive stereo camera that relies entirely on the presence of visual light. This is evident from Figure 11 which shows a mean reconstruction error between 0.085 m and 0.105 m in the presence of virtually no visible light (0.02 lx and 1.93 lx). The results show that the Realsense-Off camera is the better Realsense camera when enough light is present in the scene. In the laboratory experiments, the threshold for “enough light” is at 51 lx, and in the outdoor experiments, the threshold is found at 221 lx.

As evidenced by the plots for the mean and standard deviation, there is a significant reduction in standard deviation of the reconstruction error by the Realsense-Off camera when more light is present in the scene. The reduction is most pronounced in the laboratory setting with a decrease of 54% between 0.22 lx and 294.1 lx whereas in the outdoor setting, the reduction is 34% between 0.00 lx and 286.3 lx.

The bottom of Figure 11 shows the average number of valid depth points per depth frame from the three camera configurations. The plots support the observation that the pico flexx camera and the Realsense-On camera are insensitive to the presence of light. In the outdoor scenario, the pico flexx camera produces on average 34,900 valid depth points per frame whereas the Realsense-On camera 424,000 points per frame. The Realsense-Off camera produces 59,000 points at 0.00 lx which monotonically increase to 139,100 points at 286 lx and 206,150 points at 465 lx.

Looking at Figure 11a, the Realsense-Off camera produces significantly fewer valid depth points in the laboratory setting than in the outdoor setting; 13,350 points at 0.22 lx and 80,580 points at 294 lx in the laboratory experiments, a decrease of 77% and 42% compared to the respective illumination levels in the outdoor setting. The differences may have many causes, including the change of light source between the experiments. Another cause for the change might be the difference in materials of the interior of the sewer pipes z is noticeable in Figure 10. The pipe for the laboratory experiments consists of only one type of plastic which features a highly reflective surface whereas the interior of the outdoor pipe contains a white plastic lining with a less reflective coating. Estimation of corresponding points with a passive stereo camera is significantly harder on reflective surfaces and might thus explain some of the variation between the experiments.

#### 5.1.1. Reconstruction of the Cylindrical Pipe

The above results show the average reconstruction error with respect to the amount of light available in the scene. The following analysis will investigate how the reconstruction error is distributed throughout the extent of the cylindrical pipe. In order to show the reconstruction error of the three-dimensional pipe on two-dimensional paper, the cylinder is unfolded to the two-dimensional plane by the following equations:

(1) $$\begin{array}{cc} y_{2D} & =\theta =atan2\left(x_{3D},y_{3D}\right) \end{array}$$

(2) $$\begin{array}{cc} x_{2D} & =z_{3D} \end{array}$$

where $x_{2D}$, $y_{2D}$ denote the *x* and *y* coordinates, respectively, of the two-dimensional plane and $x_{3D}$, $y_{3D}$, and $z_{3D}$ denote the *x*, *y*, and *z*, coordinates, respectively, of the three-dimensional point cloud.

**Figure 12:**
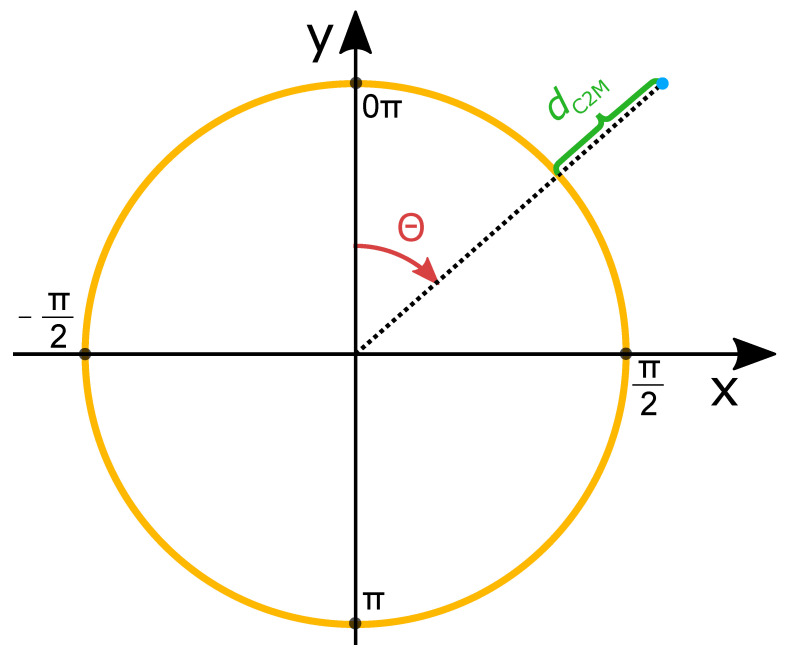
Illustration of the 3D to 2D mapping of the cylinder. The cross section of the cylinder is seen from the *XY*-plane. The angle θ is mapped onto the *y*-axis of the two-dimensional plots.

The mapping might be visualized by imagining a cylinder which is cut at the top (x=0 m, y=r) along the *z*-axis where *r* denotes the radius of the pipe. The cylinder is then unfolded to lie in the *yz*-plane. Normal convention would compute $\theta =atan2\left(y,x\right)$. However, it is deliberately chosen to compute $\theta =atan2\left(x,y\right)$ such that the origin (θ=0) is at the top of the cylinder at x=0 m, y=r. As a result of the mapping, points that do not lie directly on the cylinder are projected to the closest point on the cylindrical surface by a line that is orthogonal to the *z*-axis. The mapping is visualized in Figure 12.

**Figure 13:**
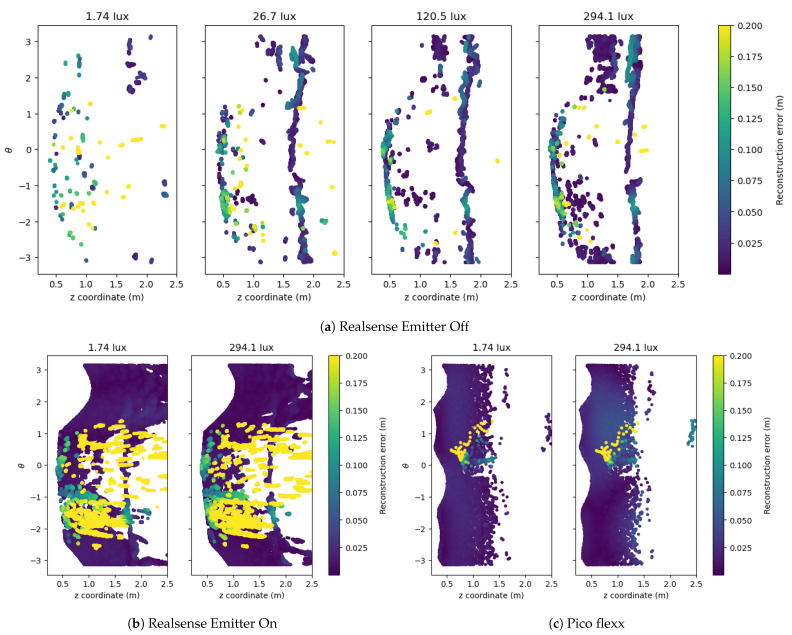
2D projection of the cloud-to-mesh distance from the three sensor configurations in the laboratory setup. The color coding of the cloud represents the cloud-to-mesh distance. If points overlay each other on the 2D projection, the point with the largest cloud-to-mesh distance is plotted on top and is thus fully visible.

**Figure 14:**
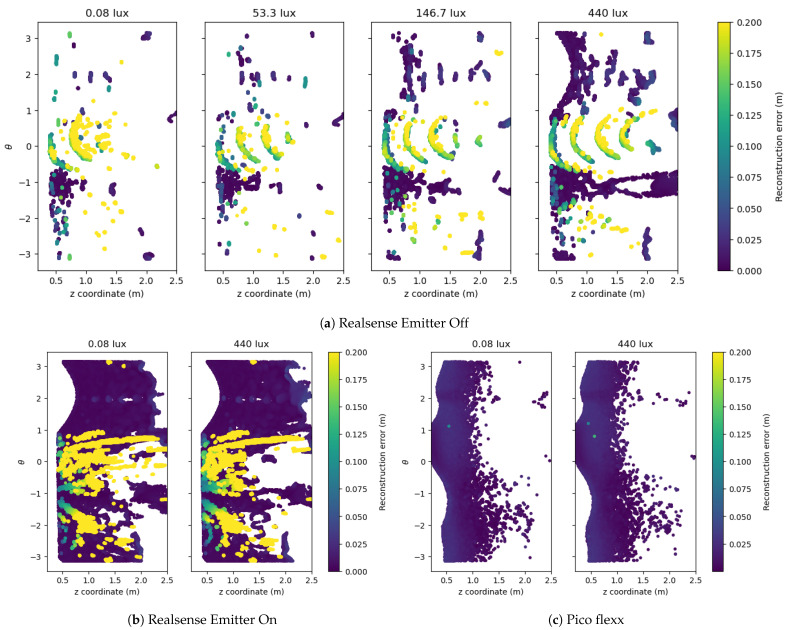
2D projection of the cloud-to-mesh distance from the three sensor configurations in the outdoor setup. The color coding of the cloud represents the cloud-to-mesh distance. If points overlay each other on the 2D projection, the point with the largest cloud-to-mesh distance is plotted on top and is thus fully visible.

The mapping of the cloud-to-mesh reconstruction error from the laboratory and outdoor experiments are shown in Figure 13 and Figure 14, respectively. The figures show the reconstruction of a single depth frame for a range of illuminance levels. The depth frames are not cherry-picked but programmatically selected from a range of frames after an initial warm-up of the depth sensors. As seen from the mean reconstruction error and the number of depth points from Figure 11, the Realsense-Off camera is the only camera configuration to be significantly affected by the absence or presence of light. For this reason, a range of four illuminance levels is visualized for the Realsense-Off camera and only two illuminance levels for the Realsense-On and pico flexx cameras, respectively.

A glance at Figure 13a and Figure 14a reveals a low, unevenly distributed point cloud of the Realsense-Off camera, which renders these point clouds useless for 3D reconstruction for robotic applications. According to Figure 11, the Realsense-Off camera produces more points for illuminance levels above 50 lx than the pico flexx camera, but the points from the Realsense-Off camera are not evenly distributed throughout the pipe. When looking at the reconstructions from Figure 13a and Figure 14a, most of the depth points seem to be located in bands either along the *z*-axis (laboratory) or along curved segments constrained along the θ-axis (outdoor).

The addition of the infrared emitter radically changes the output from the Realsense camera, producing a dense, smooth cloud of the pipe. This is apparent from the dense dark blue areas on Figure 13b and Figure 14b. The *z*-range of the cloud is longer at the bottom of the pipe (≈2 m) and shorter at the top of the pipe (≈1–5 m). However, the smooth cloud is ridden from a large number of outliers apparent as yellow sections on the 2D projections. Their errors extend far beyond the threshold on 0.2 m of the visualization and contribute to the large average error and standard deviation reported in Figure 11. These outliers are hard to filter out as they are placed randomly throughout the 3D space. Without the cropping operation performed in the post-processing step, the number of outliers would be even larger. Filtering the outliers from the Realsense-On camera is a computationally heavy operation due to the large amount of points and requires knowledge of the geometry of the pipe in order to succeed.

The point cloud from the pico flexx camera provides a consistent reconstruction with few outliers. Compared with the “good” sections of the reconstruction from the Realsense-On camera, the pico flexx point cloud shows a slightly higher error but without the many outliers of the former camera. The point cloud on the reflective laboratory pipe contains more outliers than on the matt surface of the outdoor pipe. However, the point cloud on the laboratory pipe is denser than its outdoor counterpart within the range of 0.5 m to 1.5 m.

#### 5.1.2. Reconstruction Performance Along the *z*-Axis

**Figure 15:**
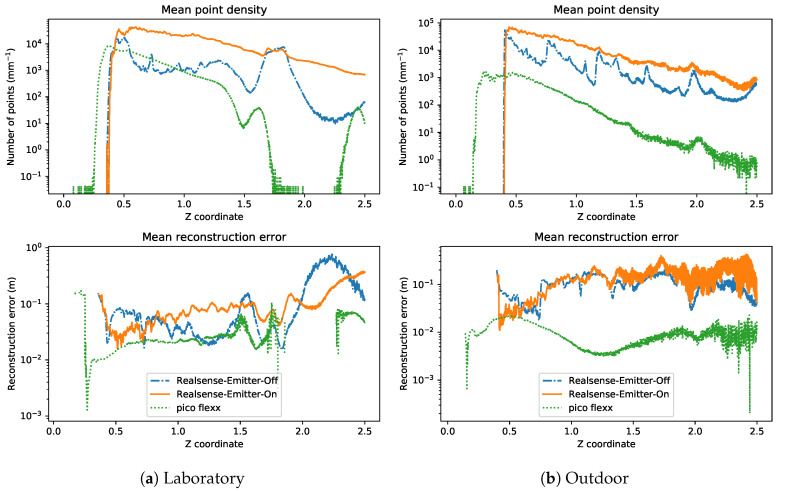
Mean point density and reconstruction error (cloud-to-mesh distance) with respect to the depth (z) coordinate of the measurement.

The performance of the point cloud may also be studied by investigating the point density and reconstruction error as a function of the depth of the pipe, e.g., the z-coordinate of the measurements. The average performance throughout all illuminance levels is shown in Figure 15.

The results from Figure 15 support the conclusions from Section 5.1.1. The pico flexx camera features a higher point density in the laboratory pipe within a range of 0.5 m to 1.5 m when compared with the outdoor pipe. However, the amount of points in the cloud above 1.5 m drops sharply in the reflective laboratory pipe whereas it gradually decreases in the outdoor pipe. At 1.5 m, however, the amount of points from the pico flexx camera drops below 10 points/mm along the *z*-axis which indicates that reconstruction beyond this depth is increasingly difficult. Looking at the reconstruction error of the pico flexx, it is observed that the camera produces a more accurate point cloud from the matt outdoor pipe than the reflective outdoor pipe. At 0.5 m, the mean reconstruction error lies at ≈0.02 m for both pipes. Above 0.5 m, the error increases slightly in the laboratory pipe but decreases in the outdoor pipe.

The dense point cloud from the Realsense-On camera starts at distance of 0.44 m with 20,000–66,000 points per mm which gradually decreases to 650–900 points per mm at the cut-off depth at 2.5 m. The graceful decrease in point density along the *z*-axis is a virtue that the active depth sensor shares with the pico flexx. The mean reconstruction error of the Realsense-On camera, however, is heavily impaired by the many outliers and does not seem to converge to a certain trend as the distance to the sensor increases.

This is also the case for the Realsense-Off camera. The point cloud from the camera provides fewer outliers than the Realsense-On camera, but the overall point cloud is so sparse that the “good” points cannot correct for the error of the outliers. The point density of the Realsense-Off camera is generally below the Realsense-On camera. In certain sections on the *z*-axis, however, the point density touches the Realsense-On camera and drops hereafter.

### 5.2. Water Level Tests

The water level tests are conducted by placing the robot just above the acrylic cover of the laboratory setup described in Section 4.4. As described in Table 4, the water level is adjusted from 0 mm to 80 mm in steps of 20 mm. The water level tests are performed with the Realsense-On and pico flexx cameras, and the post-processing of the sensor data is performed according to the instructions of Section 4.2. The point clouds are cropped such that −2 m≤x≤2 m, −2 m≤y≤2 m, and 0 m≤z≤1 m. The *z* range is limited as a consequence of the short length of the pipe used for the water level experiments.

**Figure 16:**
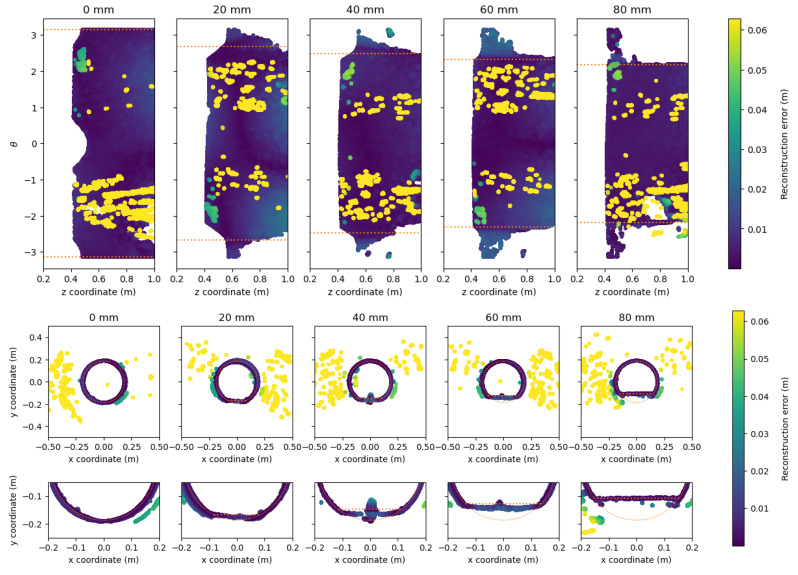
Reconstructed point cloud for the water level experiments by the Realsense-On camera. The water level and pipe geometry is indicated with the orange line. The points are color-coded according to the cloud-to-mesh distance to the reference pipe. (**Top**) unfolded view of the reconstructed pipe. The water level is indicated with an orange dotted line. (**Middle**) View from the XY-plane of the reconstructed pipe. (**Bottom**) Zoomed in view of the XY-plane of the reconstructed pipe.

**Figure 17:**
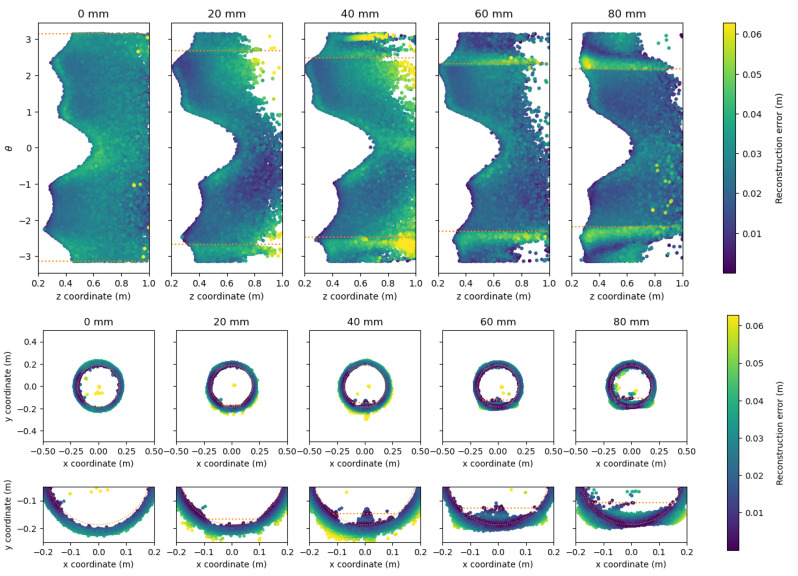
Reconstructed point cloud for the water level experiments by the pico flexx camera. The water level and pipe geometry is indicated with the orange line. The points are color-coded according to the cloud-to-mesh distance to the reference pipe. (**Top**) unfolded view of the reconstructed pipe. The water level is indicated with an orange dotted line. (**Middle**) View from the XY-plane of the reconstructed pipe. (**Bottom**) Zoomed in view of the XY-plane of the reconstructed pipe.

The reconstruction error of the point clouds is visualized by unfolding the cylindrical pipe as described in Section 5.1.1 and by showing the point cloud from the XY-plane. The reconstructions from the Realsense-On and pico flexx cameras are shown in Figure 16 and Figure 17, respectively. The water level and the geometry of the cylindrical pipe are overlaid as orange lines and orange circles, respectively, to allow for easier interpretation.

The point cloud from the Realsense-On camera approximately follows the water line, i.e., it is possible to estimate the water level by observing the lack of points where water is present in the pipe. This is evident by studying the unfolded view of the pipe and the close-up view of the XY-plane visualized in Figure 16. The dense cloud of inlier points that is close to the reference pipe is hampered by the strong presence of outliers that goes far beyond the threshold of the color scale of the cloud-to-mesh distance. The spatial extent of the outliers is noticeable from the top and middle plots of Figure 16. Consistent with the results from the clean pipes, the outliers from the Realsense-On camera are scattered randomly in 3D-space, leaving it hard to filter the point cloud to recover the actual pipe.

As opposed to the Realsense-On camera, the pico flexx camera provides a significant amount of points below the water level, rendering it difficult to estimate the water level from the cloud. When inspecting the point cloud at water levels between 40 mm to 80 mm from Figure 17, it is apparent that the cloud-to-mesh error increases slightly below the water level. The increase is seen in the bottom of Figure 17 as a slight bulge in the bottom of the point cloud. The point cloud that represents the remainder of the pipe has few outliers. When inspecting the inlier points of the cloud, the green shades of the cloud reveal that the cloud is less accurate than the inlier points of the Realsense-On camera. The slightly inaccurate cloud, however, might be a good trade-off for the sharp decrease in outliers in the water-less parts of the pipe compared with the Realsense-On camera.

## 6. Conclusions

We have compared the 3D reconstruction capabilities within the domain of sewer inspection of three different depth sensing technologies: passive stereo, active stereo, and ToF. The Realsense D435 camera is chosen as a representative of both the passive stereo and active stereo techniques as the camera is equipped with an infrared emitter that may be turned on an off; producing an active and a stereo camera, respectively. The PMD CamBoard pico flexx is chosen as a small, low-power ToF camera.

In order to compare the sensors, a laboratory and an above-ground outdoor setup were created to model confined sewer environments. The laboratory setup consists of a reflective Ø400 mm straight plastic pipe whereas the above-ground setup consists of four wells connected by Ø200 mm and Ø400 mm pipes which are used for our experiments. We have tested the capabilities of the depth sensors to reconstruct the pipes under a range of different illuminance levels; from virtually no light to 294 lx and 440 lx for the laboratory and outdoor setups, respectively. Furthermore, we have tested the performance of the ToF and active depth sensors under the presence of water in the pipe. The laboratory setup is used to simulate four different water levels, from 20 mm to 80 mm of water measured from the bottom of the pipe.

The point clouds acquired from the depth sensors are compared with 3D reference models of the pipes. The cloud-to-mesh distance between the point cloud and the 3D reference mesh is used as the main metric for assessing the reconstruction capabilities of the sensors.

The illumination tests show that both the pico flexx camera and Realsense camera with the emitter turned on (Realsense-On) are not affected by the presence nor absence of visual light. This was expected for the ToF pico flexx camera but not for the active stereo Realsense-On camera. As expected, the presence of light is instrumental for the Realsense camera with the emitter turned off (Realsense-Off). For the Realsense-Off camera, there is a clear correlation between the increase of light available in the scene and the decrease in cloud-to-mesh distance of the acquired point cloud from the sensor. Surprisingly, the Realsense-Off camera outperforms the Realsense-On counterpart when the scene is sufficiently lit. The reason for this is found in the large amount of outliers produced by the Realsense-On camera that break the otherwise dense and accurate reconstruction produced from said camera. Even though the point cloud from the Realsense-Off camera is short of the outliers that haunt the Realsense-On camera, the point cloud from the camera is sparse and unevenly distributed. This implies that the point clouds from both Realsense configurations are not suited for the purpose of 3D reconstruction in a sewer inspection context.

Compared with the Realsense-On camera, the point cloud from the pico flexx camera contains significantly fewer points than the Realsense-On camera, and in the chosen camera configuration, the amount of points beyond a distance of 1.5 m are insignificant. However, the point cloud contains few outliers and gives an accurate, dense reconstruction of the sewer pipe within a range of 0.5 to 1.5 m.

The water level tests show the same pattern with respect to the sensors; the point cloud from the Realsense-On camera is dense but noisy, and the point cloud from the pico flexx camera is less dense but more accurate. Furthermore, the tests reveal that the sensors observe the water level differently; the Realsense-On camera does not produce depth points on the water line whereas the pico flexx camera produces less accurate depth points that resembles the sections of the sewer pipe below the water line.

Overall, the comprehensive analysis of the results from the illumination tests and water level tests shows that the ToF pico flexx camera is superior to the passive and active Realsense configurations. The detrimental performance of the Realsense cameras might be caused by the reflective, featureless surfaces of the tested sewer pipes that impede the stereo matching process. However, the consistently high amount of outliers from the Realsense-On camera is a surprise. Future work could investigate if this is isolated to the specific Realsense sensor or is an issue with active stereo sensors in general. In order to utilize the data from the Realsense camera, an autonomous system must invest in heavy online post-processing of the acquired point clouds and impose strict constraints on the structure of the data, for instance, on the cylindrical shape of the pipe.

The pico flexx camera provides a compact, low-power device for depth sensing. While the spatial resolution of the depth image is significantly lower than the Realsense competitor, the camera makes up for this by providing an accurate point cloud that is unaffected by the presence or absence of light in the pipe. Overall, the pico flexx camera may be viewed as a favorite for an autonomous inspection system as the high accuracy of the point cloud reduces the need for expensive, on-board post-processing.

In this study, we have chosen to focus on depth sensing technology that provides readily available depth data with low post-processing requirements. However, one should not underestimate the importance of visual data for the purpose of detection of defects, assessment of structure, and offline validation of the system by humans. As evidenced in the literature review, there is a large body of work that focuses solely on the reconstruction of pipes from monocular and stereo imagery. The investigated depth sensors could easily be integrated with a stereo matching approach. A combined solution could offer a solution for real-time depth data and offline, detailed 3D reconstruction.

Previous works consider offline post-processing of the recorded data in order to reconstruct the pipe. If only relying on post-processing, this assumes that a human operator is able to guide the inspection vehicle as it traverses the pipe. With the exception of LiDAR and laser profilometry methods, stereo matching techniques are computationally expensive and not something that can be accommodated on a space and energy constrained inspection vehicle.

The current work investigates how recent advances in compact, solid-state depth sensing may be integrated in a future, autonomous inspection system. Aside from a simple cropping operation, the depth data is assessed “as-is”, e.g., with the assumption that the raw depth data should be readily available for tasks such as odometry, motion planning, and hazard avoidance.

The proposed work has investigated the performance of depth sensors under different lighting conditions and in the presence of dirty water in the pipe. There are, however, numerous other conditions that may occur during an inspection of sewer pipes [81] such as intruding roots, chiseled connections, blockages, leakages, and structural defects. While this study compares the sensors in non-damaged pipes, the presented results may provide directions on the performance of the depth sensors in more challenging scenarios.

## Figures and Tables

**Table 1:** Overview of related work on depth sensing technology for internal inspection of sewers or pipe networks. The depth sensing hardware of each paper is categorized into four categories; *Camera* if the setup contains any camera, *Stereo* if the setup contains a passive or active stereo setup, *Laser* if the setup contains a one-dimensional (rotating) laser, and *omnidirectional* if the setup either contains a catadioptric sensor or a camera with a fish-eye lens.

Paper	Year	Camera	Stereo	Laser	Omni-Directional	Other Sensors	Reconstruction Technique
[14]	1995			X			Laser profilometry
[13,15]	1998	X		X		Ultras.	Laser profilometry
[16]	1998	X		X			Laser profilometry
[17]	1999	X	X				None
[18]	2000	X					Image proc. + Hough transform
[19]	2000	X		X			Image proc. + Hough transform
[20]	2002	X	X				None
[21]	2003					LiDAR	ICP
[22]	2003	X					Laser profilometry
[23]	2005	X		X	X		Laser profilometry
[24]	2007			X			Laser profilometry
[9]	2007	X		X	X		Laser profilometry
[25]	2007	X			X		SfM + cylinder fitting
[26]	2008	X		X			Laser profilometry
[27]	2009	X					Sparse 3D + mosaicing
[28,29]	2009	X			X		SfM
[30]	2010	X	X	X		Ultras.	Image proc. + feat. match.
[31]	2011	X	X				Dense stereo matching
[32]	2011	X			X		Dense stereo matching
[33]	2013			X			Laser profilometry
[34]	2013	X		X			Laser profilometry
[35]	2013	X		X			Laser profilometry
[36]	2014	X				LiDAR	ICP
[37]	2014	X		X			Structured light
[38]	2014	X		X			Dense stereo matching
[39]	2014	X					Fourier image correspond.
[40]	2014			X			Commercial laser scanner
[41]	2014	X				ToF	Hough transf. (cylinder)
[42]	2015	X	X			Kinect	RGB-D SLAM
[43]	2015	X		X			Structured light + sparse 3D
[44]	2015	X	X	X	X		Laser profilometry
[45]	2015	X	X				Dense stereo matching
[46]	2016	X	X				Dense stereo matching
[47]	2016	X		X			Laser profilometry
[48]	2017	X		X			Laser profilometry
[49]	2017	X	X			LiDAR	Dense stereo matching
[50]	2018	X	X	X	X		Laser profilometry
[51]	2018	X	X			ToF	Visual inertial odometry
[52]	2019	X					Dense stereo matching
[53]	2019	X			X		Dense stereo matching + SfM
[54]	2019	X		X			Laser profilometry
[55]	2019	X		X	X		Laser profilometry
[56]	2019	X					Dense stereo matching
[57]	2019	X				LiDAR	Radius estimation from curvature
[58]	2019	X			X		Dense stereo matching
[59]	2020	X	X	X		LiDAR	RGB-D SLAM
[60]	2020	X	X			Kinect	ICP + visual SLAM
[61]	2020	X	X			LiDAR	ICP
[62]	2020	X	X				Dense stereo matching

**Table 2:** Specifications of the chosen pico flexx [69] and RealSense D435 cameras [70].

Camera	Resolution (Pixels)	Frame Rate (fps)	Range (m)	Power (w)	Size (mm)	Weight (g)
PMD CamBoard pico flexx	224 × 171	45	0.1–4	≈0.3	68 × 17 × 7.25	8
RealSense D435	1920 × 1080	30	0.3–3	<2	90 × 25 × 25	9

**Table 3:** Configuration of the experimental setups for the illumination tests.

Test Setup	Pipe	Light Source	Illuminance Levels		# Levels
			Low	High	
Lab	Ø400 PVC	Custom ring light	0.02 lx	294 lx	23
Outdoor	Ø400 PVC	Smart Vision Lights S75-Whi [71]	0.08 lx	440 lx	9

**Table 4:** Details of the pollutants added to the laboratory water for the water level experiments. The amount of pollutants are found experimentally to maintain a certain level of cloudiness in the water.

Water Level	Amount of Ground Coffee	Amount of Milk
0 mm	0 g	0.0%
20 mm	35 g	0.5%
40 mm	97 g	0.2%
60 mm	97 g	0.5%
80 mm	270 g	0.5%

## Data Availability

Not applicable.

## Abbreviations

The following abbreviations are used in this manuscript:

C2M	Cloud-to-mesh
CCD	Charged Coupled Device
CCTV	Closed-Circuit Television
CMOS	Complementary metal–oxide–semiconductor
GNSS	Global Navigation Satellite System
ICP	Iterative Closest Points
IMU	Inertial measurement unit
SfM	Structure-from-motion
SLAM	Simultaneous Localization and Mapping
TOF	Time-of-flight
